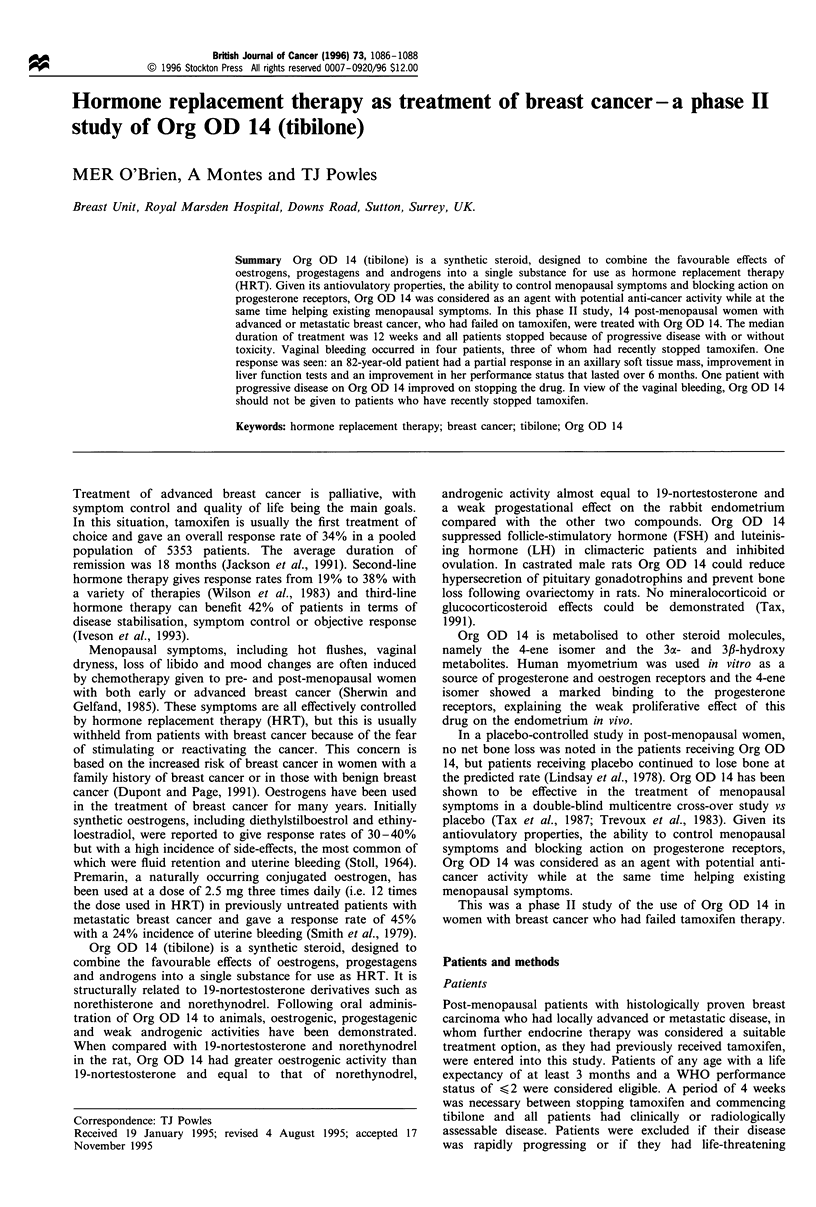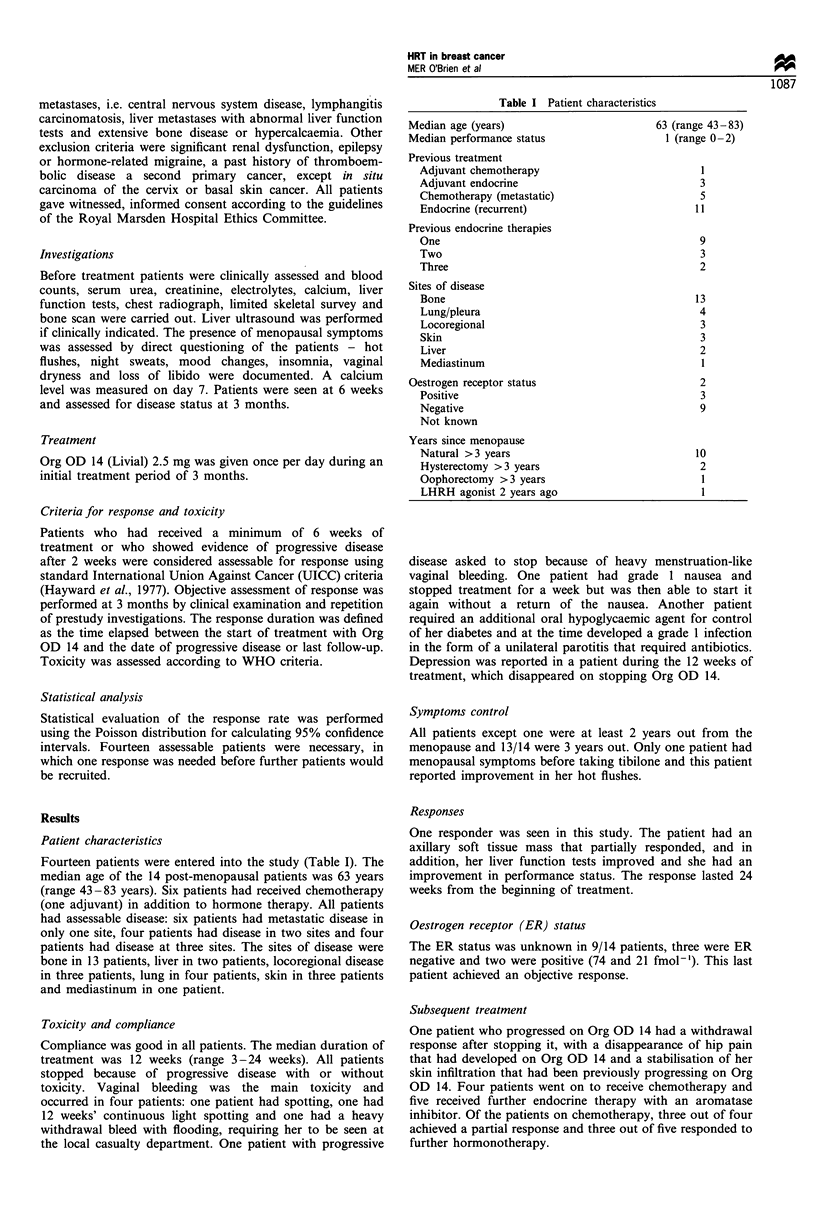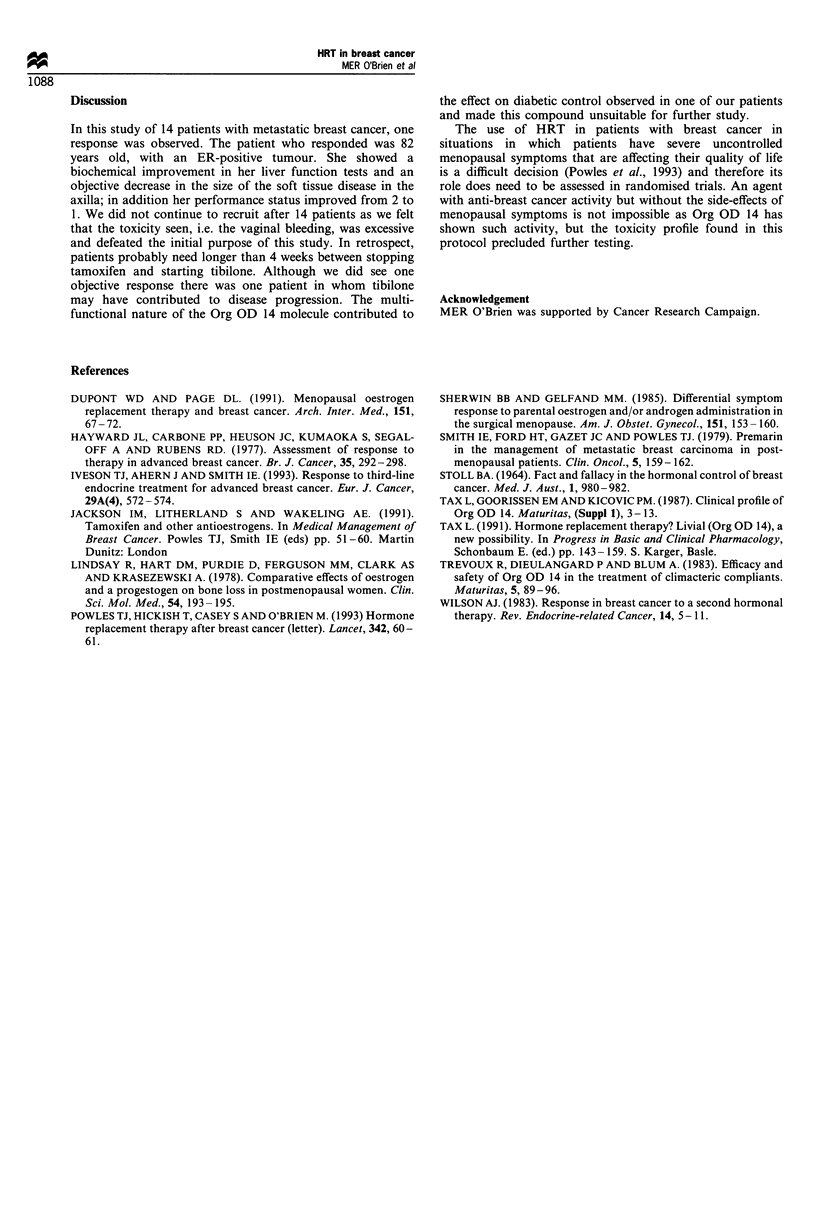# Hormone replacement therapy as treatment of breast cancer--a phase II study of Org OD 14 (tibilone).

**DOI:** 10.1038/bjc.1996.209

**Published:** 1996-05

**Authors:** M. O'Brien, A. Montes, T. J. Powles

**Affiliations:** Breast Unit, Royal Marsden Hospital, Sutton, Surrey, UK.

## Abstract

Org OD 14 (tibilone) is a synthetic steroid, designed to combine the favourable effects of oestrogens, progestagens and androgens into a single substance for use as hormone replacement therapy (HRT). Given its antiovulatory properties, the ability to control menopausal symptoms and blocking action on progesterone receptors, Org OD 14 was considered as an agent with potential anti-cancer activity while at the same time helping existing menopausal symptoms. In this phase II study, 14 post-menopausal women with advanced or metastatic breast cancer, who had failed on tamoxifen, were treated with Org OD 14. The median duration of treatment was 12 weeks and all patients stopped because of progressive disease with or without toxicity. Vaginal bleeding occurred in four patients, three of whom had recently stopped tamoxifen. One response was seen: an 82-year-old patient had a partial response in an axillary soft tissue mass, improvement in liver function tests and an improvement in her performance status that lasted over 6 months. One patient with progressive disease on Org OD 14 improved on stopping the drug. In view of the vaginal bleeding, Org OD 14 should not be given to patients who have recently stopped tamoxifen.


British Journal of Cancer (1996) 73, 1086-1088
© 1996 Stockton Press All rights reserved 0007-0920/96 $12.00

### Hormone replacement therapy as treatment of breast cancer – a phase II study of Org OD 14 (tibilone)

MER O'Brien, A Montes and TJ Powles

*Breast Unit, Royal Marsden Hospital, Downs Road, Sutton, Surrey, UK.*

**Summary** Org OD 14 (tibilone) is a synthetic steroid, designed to combine the favourable effects of oestrogens, progestagens and androgens into a single substance for use as hormone replacement therapy (HRT). Given its antiovulatory properties, the ability to control menopausal symptoms and blocking action on progesterone receptors, Org OD 14 was considered as an agent with potential anti-cancer activity while at the same time helping existing menopausal symptoms. In this phase II study, 14 post-menopausal women with advanced or metastatic breast cancer, who had failed on tamoxifen, were treated with Org OD 14. The median duration of treatment was 12 weeks and all patients stopped because of progressive disease with or without toxicity. Vaginal bleeding occurred in four patients, three of whom had recently stopped tamoxifen. One response was seen: an 82-year-old patient had a partial response in an axillary soft tissue mass, improvement in liver function tests and an improvement in her performance status that lasted over 6 months. One patient with progressive disease on Org OD 14 improved on stopping the drug. In view of the vaginal bleeding, Org OD 14 should not be given to patients who have recently stopped tamoxifen.

**Keywords:** hormone replacement therapy; breast cancer; tibilone; Org OD 14

Treatment of advanced breast cancer is palliative, with symptom control and quality of life being the main goals. In this situation, tamoxifen is usually the first treatment of choice and gave an overall response rate of 34% in a pooled population of 5353 patients. The average duration of remission was 18 months (Jackson *et al.*, 1991). Second-line hormone therapy gives response rates from 19% to 38% with a variety of therapies (Wilson *et al.*, 1983) and third-line hormone therapy can benefit 42% of patients in terms of disease stabilisation, symptom control or objective response (Iveson *et al.*, 1993).

Menopausal symptoms, including hot flushes, vaginal dryness, loss of libido and mood changes are often induced by chemotherapy given to pre- and post-menopausal women with both early or advanced breast cancer (Sherwin and Gelfand, 1985). These symptoms are all effectively controlled by hormone replacement therapy (HRT), but this is usually withheld from patients with breast cancer because of the fear of stimulating or reactivating the cancer. This concern is based on the increased risk of breast cancer in women with a family history of breast cancer or in those with benign breast cancer (Dupont and Page, 1991). Oestrogens have been used in the treatment of breast cancer for many years. Initially synthetic oestrogens, including diethylstilboestrol and ethinyloestradiol, were reported to give response rates of 30–40% but with a high incidence of side-effects, the most common of which were fluid retention and uterine bleeding (Stoll, 1964). Premarin, a naturally occurring conjugated oestrogen, has been used at a dose of 2.5 mg three times daily (i.e. 12 times the dose used in HRT) in previously untreated patients with metastatic breast cancer and gave a response rate of 45% with a 24% incidence of uterine bleeding (Smith *et al.*, 1979).

Org OD 14 (tibilone) is a synthetic steroid, designed to combine the favourable effects of oestrogens, progestagens and androgens into a single substance for use as HRT. It is structurally related to 19-nortestosterone derivatives such as norethisterone and norethynodrel. Following oral administration of Org OD 14 to animals, oestrogenic, progestagenic and weak androgenic activities have been demonstrated. When compared with 19-nortestosterone and norethynodrel in the rat, Org OD 14 had greater oestrogenic activity than 19-nortestosterone and equal to that of norethynodrel, androgenic activity almost equal to 19-nortestosterone and a weak progestational effect on the rabbit endometrium compared with the other two compounds. Org OD 14 suppressed follicle-stimulatory hormone (FSH) and luteinising hormone (LH) in climacteric patients and inhibited ovulation. In castrated male rats Org OD 14 could reduce hypersecretion of pituitary gonadotrophins and prevent bone loss following ovariectomy in rats. No mineralocorticoid or glucocorticosteroid effects could be demonstrated (Tax, 1991).

Org OD 14 is metabolised to other steroid molecules, namely the 4-ene isomer and the 3α- and 3β-hydroxy metabolites. Human myometrium was used *in vitro* as a source of progesterone and oestrogen receptors and the 4-ene isomer showed a marked binding to the progesterone receptors, explaining the weak proliferative effect of this drug on the endometrium *in vivo*.

In a placebo-controlled study in post-menopausal women, no net bone loss was noted in the patients receiving Org OD 14, but patients receiving placebo continued to lose bone at the predicted rate (Lindsay *et al.*, 1978). Org OD 14 has been shown to be effective in the treatment of menopausal symptoms in a double-blind multicentre cross-over study *vs* placebo (Tax *et al.*, 1987; Trevoux *et al.*, 1983). Given its antiovulatory properties, the ability to control menopausal symptoms and blocking action on progesterone receptors, Org OD 14 was considered as an agent with potential anti-cancer activity while at the same time helping existing menopausal symptoms.

This was a phase II study of the use of Org OD 14 in women with breast cancer who had failed tamoxifen therapy.

### Patients and methods

### *Patients*

Post-menopausal patients with histologically proven breast carcinoma who had locally advanced or metastatic disease, in whom further endocrine therapy was considered a suitable treatment option, as they had previously received tamoxifen, were entered into this study. Patients of any age with a life expectancy of at least 3 months and a WHO performance status of ≤2 were considered eligible. A period of 4 weeks was necessary between stopping tamoxifen and commencing tibilone and all patients had clinically or radiologically assessable disease. Patients were excluded if their disease was rapidly progressing or if they had life-threatening

Correspondence: TJ Powles
Received 19 January 1995; revised 4 August 1995; accepted 17 November 1995

metastases, i.e. central nervous system disease, lymphangitis carcinomatosis, liver metastases with abnormal liver function tests and extensive bone disease or hypercalcaemia. Other exclusion criteria were significant renal dysfunction, epilepsy or hormone-related migraine, a past history of thromboembolic disease a second primary cancer, except *in situ* carcinoma of the cervix or basal skin cancer. All patients gave witnessed, informed consent according to the guidelines of the Royal Marsden Hospital Ethics Committee.

### *Investigations*

Before treatment patients were clinically assessed and blood counts, serum urea, creatinine, electrolytes, calcium, liver function tests, chest radiograph, limited skeletal survey and bone scan were carried out. Liver ultrasound was performed if clinically indicated. The presence of menopausal symptoms was assessed by direct questioning of the patients – hot flushes, night sweats, mood changes, insomnia, vaginal dryness and loss of libido were documented. A calcium level was measured on day 7. Patients were seen at 6 weeks and assessed for disease status at 3 months.

### *Treatment*

Org OD 14 (Livial) 2.5 mg was given once per day during an initial treatment period of 3 months.

### *Criteria for response and toxicity*

Patients who had received a minimum of 6 weeks of treatment or who showed evidence of progressive disease after 2 weeks were considered assessable for response using standard International Union Against Cancer (UICC) criteria (Hayward *et al*., 1977). Objective assessment of response was performed at 3 months by clinical examination and repetition of prestudy investigations. The response duration was defined as the time elapsed between the start of treatment with Org OD 14 and the date of progressive disease or last follow-up. Toxicity was assessed according to WHO criteria.

### *Statistical analysis*

Statistical evaluation of the response rate was performed using the Poisson distribution for calculating 95% confidence intervals. Fourteen assessable patients were necessary, in which one response was needed before further patients would be recruited.

### Results

### *Patient characteristics*

Fourteen patients were entered into the study (Table I). The median age of the 14 post-menopausal patients was 63 years (range 43–83 years). Six patients had received chemotherapy (one adjuvant) in addition to hormone therapy. All patients had assessable disease: six patients had metastatic disease in only one site, four patients had disease in two sites and four patients had disease at three sites. The sites of disease were bone in 13 patients, liver in two patients, locoregional disease in three patients, lung in four patients, skin in three patients and mediastinum in one patient.

### *Toxicity and compliance*

Compliance was good in all patients. The median duration of treatment was 12 weeks (range 3–24 weeks). All patients stopped because of progressive disease with or without toxicity. Vaginal bleeding was the main toxicity and occurred in four patients: one patient had spotting, one had 12 weeks' continuous light spotting and one had a heavy withdrawal bleed with flooding, requiring her to be seen at the local casualty department. One patient with progressive disease asked to stop because of heavy menstruation-like vaginal bleeding. One patient had grade 1 nausea and stopped treatment for a week but was then able to start it again without a return of the nausea. Another patient required an additional oral hypoglycaemic agent for control of her diabetes and at the time developed a grade 1 infection in the form of a unilateral parotitis that required antibiotics. Depression was reported in a patient during the 12 weeks of treatment, which disappeared on stopping Org OD 14.

**Table I** Patient characteristics

| | |
|---|---|
| Median age (years) | 63 (range 43–83) |
| Median performance status | 1 (range 0–2) |
| Previous treatment | |
| Adjuvant chemotherapy | 1 |
| Adjuvant endocrine | 3 |
| Chemotherapy (metastatic) | 5 |
| Endocrine (recurrent) | 11 |
| Previous endocrine therapies | |
| One | 9 |
| Two | 3 |
| Three | 2 |
| Sites of disease | |
| Bone | 13 |
| Lung/pleura | 4 |
| Locoregional | 3 |
| Skin | 3 |
| Liver | 2 |
| Mediastinum | 1 |
| Oestrogen receptor status | 2 |
| Positive | 3 |
| Negative | 9 |
| Not known | |
| Years since menopause | |
| Natural >3 years | 10 |
| Hysterectomy >3 years | 2 |
| Oophorectomy >3 years | 1 |
| LHRH agonist 2 years ago | 1 |

### *Symptoms control*

All patients except one were at least 2 years out from the menopause and 13/14 were 3 years out. Only one patient had menopausal symptoms before taking tibilone and this patient reported improvement in her hot flushes.

### *Responses*

One responder was seen in this study. The patient had an axillary soft tissue mass that partially responded, and in addition, her liver function tests improved and she had an improvement in performance status. The response lasted 24 weeks from the beginning of treatment.

### *Oestrogen receptor (ER) status*

The ER status was unknown in 9/14 patients, three were ER negative and two were positive (74 and 21 $fmol^{-1}$). This last patient achieved an objective response.

### *Subsequent treatment*

One patient who progressed on Org OD 14 had a withdrawal response after stopping it, with a disappearance of hip pain that had developed on Org OD 14 and a stabilisation of her skin infiltration that had been previously progressing on Org OD 14. Four patients went on to receive chemotherapy and five received further endocrine therapy with an aromatase inhibitor. Of the patients on chemotherapy, three out of four achieved a partial response and three out of five responded to further hormonotherapy.

### Discussion

In this study of 14 patients with metastatic breast cancer, one response was observed. The patient who responded was 82 years old, with an ER-positive tumour. She showed a biochemical improvement in her liver function tests and an objective decrease in the size of the soft tissue disease in the axilla; in addition her performance status improved from 2 to 1. We did not continue to recruit after 14 patients as we felt that the toxicity seen, i.e. the vaginal bleeding, was excessive and defeated the initial purpose of this study. In retrospect, patients probably need longer than 4 weeks between stopping tamoxifen and starting tibilone. Although we did see one objective response there was one patient in whom tibilone may have contributed to disease progression. The multifunctional nature of the Org OD 14 molecule contributed to the effect on diabetic control observed in one of our patients and made this compound unsuitable for further study.

The use of HRT in patients with breast cancer in situations in which patients have severe uncontrolled menopausal symptoms that are affecting their quality of life is a difficult decision (Powles *et al.*, 1993) and therefore its role does need to be assessed in randomised trials. An agent with anti-breast cancer activity but without the side-effects of menopausal symptoms is not impossible as Org OD 14 has shown such activity, but the toxicity profile found in this protocol precluded further testing.

**Acknowledgement**

MER O'Brien was supported by Cancer Research Campaign.

### References

DUPONT WD AND PAGE DL. (1991). Menopausal oestrogen replacement therapy and breast cancer. *Arch. Inter. Med.*, **151**, 67–72.

HAYWARD JL, CARBONE PP, HEUSON JC, KUMAOKA S, SEGALOFF A AND RUBENS RD. (1977). Assessment of response to therapy in advanced breast cancer. *Br. J. Cancer*, **35**, 292–298.

IVESON TJ, AHERN J AND SMITH IE. (1993). Response to third-line endocrine treatment for advanced breast cancer. *Eur. J. Cancer*, **29A(4)**, 572–574.

JACKSON IM, LITHERLAND S AND WAKELING AE. (1991). Tamoxifen and other antioestrogens. In *Medical Management of Breast Cancer*. Powles TJ, Smith IE (eds) pp. 51–60. Martin Dunitz: London

LINDSAY R, HART DM, PURDIE D, FERGUSON MM, CLARK AS AND KRASEZEWSKI A. (1978). Comparative effects of oestrogen and a progestogen on bone loss in postmenopausal women. *Clin. Sci. Mol. Med.*, **54**, 193–195.

POWLES TJ, HICKISH T, CASEY S AND O'BRIEN M. (1993) Hormone replacement therapy after breast cancer (letter). *Lancet*, **342**, 60–61.

SHERWIN BB AND GELFAND MM. (1985). Differential symptom response to parental oestrogen and/or androgen administration in the surgical menopause. *Am. J. Obstet. Gynecol.*, **151**, 153–160.

SMITH IE, FORD HT, GAZET JC AND POWLES TJ. (1979). Premarin in the management of metastatic breast carcinoma in postmenopausal patients. *Clin. Oncol.*, **5**, 159–162.

STOLL BA. (1964). Fact and fallacy in the hormonal control of breast cancer. *Med. J. Aust.*, **1**, 980–982.

TAX L, GOORISSEN EM AND KICOVIC PM. (1987). Clinical profile of Org OD 14. *Maturitas*, **(Suppl 1)**, 3–13.

TAX L. (1991). Hormone replacement therapy? Livial (Org OD 14), a new possibility. In *Progress in Basic and Clinical Pharmacology*, Schonbaum E. (ed.) pp. 143–159. S. Karger, Basle.

TREVOUX R, DIEULANGARD P AND BLUM A. (1983). Efficacy and safety of Org OD 14 in the treatment of climacteric compliants. *Maturitas*, **5**, 89–96.

WILSON AJ. (1983). Response in breast cancer to a second hormonal therapy. *Rev. Endocrine-related Cancer*, **14**, 5–11.